# Modulation of Multidrug Resistance Protein 1-mediated Transport Processes by the Antiviral Drug Ritonavir in Cultured Primary Astrocytes

**DOI:** 10.1007/s11064-023-04008-5

**Published:** 2023-08-21

**Authors:** Christian Arend, Isabell L. Grothaus, Mario Waespy, Lucio Colombi Ciacchi, Ralf Dringen

**Affiliations:** 1 Centre for Biomolecular Interactions Bremen, Faculty 2 (Biology/Chemistry), University of Bremen, P.O. Box 330440, 28359 Bremen, Germany; 2 Centre for Environmental Research and Sustainable Technology, University of Bremen, Bremen, Germany; 3 Hybrid Materials Interfaces Group, Faculty of Production Engineering, Bremen Center for Computational Materials Science, MAPEX Center for Materials and Processes, University of Bremen, Am Fallturm 1, 28359 Bremen, Germany

**Keywords:** Astrocyte, Glutathione, Molecular Dynamics Simulation, Mrp1, Ritonavir¸ Transport

## Abstract

The Multidrug Resistance Protein 1 (Mrp1) is an ATP-dependent efflux transporter and a major facilitator of drug resistance in mammalian cells during cancer and HIV therapy. In brain, Mrp1-mediated GSH export from astrocytes is the first step in the supply of GSH precursors to neurons. To reveal potential mechanisms underlying the drug-induced modulation of Mrp1-mediated transport processes, we investigated the effects of the antiviral drug ritonavir on cultured rat primary astrocytes. Ritonavir strongly stimulated the Mrp1-mediated export of glutathione (GSH) by decreasing the K_m_ value from 200 nmol/mg to 28 nmol/mg. In contrast, ritonavir decreased the export of the other Mrp1 substrates glutathione disulfide (GSSG) and bimane-glutathione. To give explanation for these apparently contradictory observations, we performed *in silico* docking analysis and molecular dynamics simulations using a homology model of rat Mrp1 to predict the binding modes of ritonavir, GSH and GSSG to Mrp1. The results suggest that ritonavir binds to the hydrophilic part of the bipartite binding site of Mrp1 and thereby differently affects the binding and transport of the Mrp1 substrates. These new insights into the modulation of Mrp1-mediated export processes by ritonavir provide a new model to better understand GSH-dependent detoxification processes in brain cells.

**Supplementary Information:**

The online version contains supplementary material available at 10.1007/s11064-023-04008-5.

## Introduction

The Multidrug Resistance Protein 1 (Mrp1) was firstly discovered in 1992 in a drug-resistant human lung-cancer cell-line and is encoded by the ABCC1 gene [1, 2]. Mrp1 belongs to the family of ATP-binding cassette (ABC) proteins and is, with the exception of the liver, ubiquitously expressed within the body [1, 3–5]. The ability of Mrp1 to effectively export xenobiotic substances, especially drugs, is clinically highly relevant due to the elevated expression levels of Mrp1 in a variety of drug-resistant tumour tissues [1, 4, 6, 7]. For example, the inhibition of Mrp1 in astrocytoma tumor cells significantly increases the response of these cells to chemotherapeutic drugs like etoposide and vincristine [8]. This underlies the importance of Mrp1 as a significant target to improve anti-cancer treatment. However, also in normal cells Mrp1 is essential for cellular metabolism, survival and communication by mediating the cellular efflux of a broad range of substrates, such as reduced (GSH) or oxidised (GSSG) glutathione, and a variety of organic anions including their conjugates to GSH (e.g. leukotriene C4 (LTC4)), glucuronide or sulfate [1, 9–11]. However, mechanisms underlying the transport processes of these numerous cargos are poorly understood. But since the molecular structure of bovine Mrp1 had already been resolved using cryo-electron microscopy, an in-depth view into molecular details of the complete transport mechanism was possible [12, 13]. Because of the immense physiological and pharmacological importance of Mrp1, there is increasing interest to understand its substrate-dependent transport mechanism and to identify modulators of Mrp1 activity and function [5, 14].

Primary astrocyte cultures have been extensively used as model systems to study Mrp1-dependent transport processes, and substantial information has been published concerning the export of the physiological Mrp1 substrates GSH and GSSG [15–18]. Furthermore, these primary cultures possess an obvious advantage over tumor cell-based model systems, since they more closely reflect the in vivo situation. Indeed, no artificial overexpression (especially of Mrp1) or cell-line-based alterations in endogenous expression levels have to be taken into consideration. Astrocytes are not only the most abundant glial cell type in brain [19]; they also play a pivotal role in the brain’s normal and drug-related metabolism [20–22]. Therefore, astrocytes are considered as an emerging therapeutic target in several neurological disorders including Alzheimer’s Disease [23–25]. Most importantly, astrocytes possess a strategically exceptional position within the brain, as they cover brain capillaries nearly completely with their endfeet and are therefore the first parenchymal brain cell type that encounters substances which are delivered by the bloodstream and cross the blood-brain barrier [26]. On the other hand, astrocytes are tightly associated with neurons and their synapses [27, 28]. Thereby, astrocytes maintain synaptic connectivity by taking up neurotransmitters from the synaptic cleft [27, 28]. In addition, astrocytes supply neurons with energy substrates in the form of lactate that is derived from glycolysis and/or glycogen mobilization [29, 30] and with precursors for GSH synthesis [31, 32].

Among other ABC-family members, Mrp1 is expressed in astrocyte primary cultures [16, 18, 33, 34] and in astrocytes in brain on mRNA and protein level [35, 36]. For these cells, Mrp1 has been shown to mediate the export of GSH [15, 18], GSSG [15, 16, 18, 37] and bimane-glutathione (GS-B) [37, 38]. Furthermore, Mrp1 has been shown to be predominantly responsible for the export of GSH and exclusively responsible for that of GSSG in primary astrocytes [18]. The export of these three Mrp1 substrates from astrocytes has been reported to be efficiently inhibited by the Mrp1 inhibitor MK571 [16–18, 37, 38]. Previous studies have demonstrated that Mrp1 mediates 60% of the GSH export from primary astrocytes of mouse [18] and rat origin [17]. The remaining 40% of GSH export has so far not been attributed to a specific transporter and contributions of Mrp4, Mrp5, cystic fibrosis transmembrane transductance regulator or organic anion transporting polypeptides 1 or 2 in GSH export appear to be unlikely [18]. However, since the Mrp1-independent part of GSH export in Mrp1 knock-out astrocytes was not affected by the presence of MK571, the MK571-inhibitable GSH export from astrocytes is considered to represent exclusively Mrp1-mediated export [18].

This astroglial GSH export is essential to supply precursors for GSH synthesis to neighbouring neurons [31, 32, 39]. During oxidative stress GSH is oxidised to glutathione disulfide (GSSG) [16, 37] to provide electrons for the reduction of reactive oxygen species (ROS) [39, 40]. Subsequently, GSSG is rapidly exported from cultured astrocytes, which slows down the rapid oxidation of the cellular thiol reduction potential [39, 40]. Additionally, GSH plays an essential role in the detoxification of organic compounds, as it can be conjugated to various types of xenobiotics via glutathione-*S*-transferases to dispose potentially harmful substances that are subsequently exported from the cells via Mrp1 [39, 41, 42].

Since the mid 90’s, infection with the human immunodeficiency virus (HIV) is efficiently treated by the combination of different classes of antiretroviral drugs [43, 44]. One class of such drugs are inhibitors of the HIV protease, which prevent the maturation of viral proteins by the inhibition of viral replication [45, 46]. Ritonavir is one of the first prescribed HIV-protease inhibitors and was approved by the FDA in 1996 [46, 47]. Ritonavir is nowadays used primarily to increase the bioavailability of other HIV drugs as it inhibits their major metabolising enzyme, P450 3A4 monooxygenase [48, 49]. For the latter reason, ritonavir is used in combination with the COVID-19 protease inhibitor nirmatrelvir as part of the new therapeutic drug paxlovid, which has been shown to drastically reduce the risk of severe disease progression after infection with COVID-19 [50, 51].

Furthermore, ritonavir has been shown to stimulate the GSH export from cultured astrocytes and this stimulated GSH export in presence of ritonavir can be completely blocked by MK571 [52]. However, the mechanism involved in this stimulation has not been reported so far. Here we demonstrate that incubation with ritonavir lowers the K_m_ value for the Mrp1-mediated GSH export from viable astrocytes, while the V_max_ value remains unaltered. Furthermore, higher concentrations of ritonavir lower the export of the Mrp1 substrates GSSG and GS-B from viable astrocytes. *In silico* docking and molecular dynamics (MD) simulation of bound ritonavir, GSH and/or GSSG to a homology model of rat Mrp1 confirmed that ritonavir is likely to bind to the hydrophilic part of the bipartite Mrp1 substrate binding site, thereby diversely affecting the binding and transport of different Mrp1 substrates.

## Experimental Procedures

### Materials

Ritonavir and menadione were purchased from Sigma-Aldrich (Steinheim, Germany) and MK571 from Biomol (Hamburg, Germany). Monochlorobimane (MCB) was from Fluka (Buchs, Switzerland). Dulbecco’s modified Eagle’s medium (DMEM containing 25 mM glucose) was from Gibco (Darmstadt, Germany), fetal calf serum was obtained from Biochrom (Berlin, Germany) and penicillin/streptomycin solution from Invitrogen-Gibco (Darmstadt, Germany). Sulfosalicylic acid (SSA), bovine serum albumin (BSA), NADPH and NADH were bought from Applichem (Darmstadt, Germany). The enzyme glutathione reductase was purchased from Roche Diagnostics (Mannheim, Germany). All other chemicals of the highest purity available were obtained from Merck (Darmstadt, Germany), Sigma-Aldrich (Steinheim, Germany), Fluka (Buchs, Switzerland), Roth (Karlsruhe, Germany) or Riedel-de-Haën (Seelze, Germany). Sterile cell culture plates, unsterile 96-well microtiter plates and unsterile black 96-well microtiter plates were from Sarstedt (Nümbrecht, Germany).

### Astrocyte Cultures

Astrocyte-rich primary cultures were prepared from the brains of newborn Wistar rats as described in detail previously [53]. 300 000 viable cells were seeded in 1 mL culture medium (90% DMEM containing 25 mM glucose, 20 U/mL penicillin G, 20 µg/mL streptomycin, 44.6 mM NaHCO_3_ and 1 mM pyruvate supplemented with 10% fetal calf serum) into wells of 24-well plates. The cultures were incubated in a Sanyo incubator (Osaka, Japan) with 10% CO_2_ at 37 °C in a humidified atmosphere. Every 7 days and 24 h prior to the experiments, the culture medium was renewed. The export experiments of the present study were performed on confluent cultures of an age between 21 and 35 days. These cultures contain mainly astrocytes and only low amounts of contaminating oligodendrocytes and microglial cells [53, 54].

### Experimental Incubation of the Cells

For export studies, the cultures were washed twice with 1 mL prewarmed (37 °C) incubation buffer (IB; 20 mM HEPES, 5 mM D-glucose, 145 mM NaCl, 5.4 mM KCl, 1.8 mM CaCl_2_, 1 mM MgCl_2_, 0.8 mM Na_2_HPO_4_, pH adjusted with NaOH at 37 °C to 7.4) and afterwards incubated at 37 °C with 200 µL (all experiments without MCB) or 500 µL (all experiments containing MCB) IB containing 100 µM of the γ-glutamyltranspeptidase inhibitor acivicin [55] in the absence or the presence of the compounds indicated in the figures and the table. The incubation media were collected after the given incubation periods, and the cells were washed with 1 mL ice-cold phosphate-buffered saline (PBS; 10 mM potassium phosphate buffer, pH 7.4, containing 150 mM NaCl) before the extracellular and cellular GSx (GSx = amount of GSH plus twice the amount of GSSG) and GSSG contents were determined.

To lower the initial cellular GSx content to different extents in order to perform kinetic GSH export studies, the cells were preincubated in the absence or presence of 100 µM of the GSH synthesis inhibitor L-buthionine sulfoximine (BSO) in 1 mL DMEM for different timepoints of up to 24 h at 37 °C. Afterwards, the medium was aspirated and the cells were washed twice with 1 mL prewarmed (37 °C) IB. Subsequently, the cells were further incubated at 37 °C in 200 µL IB containing 100 µM acivicin in the absence (control) or presence of 10 µM ritonavir. Ten µL media samples were taken every 30 min for up to 180 min (control) or every 10 min for up to 60 min (presence of ritonavir).

Cultured astrocytes oxidize GSH to GSSG during oxidative stress, which is subsequently exported via Mrp1 [16, 18]. In the current study, cellular oxidation of GSH to GSSG was induced by application of menadione [37, 56]. Cultured astrocytes were washed twice with 1 mL prewarmed (37 °C) IB and afterwards incubated with 200 µL of IB containing 100 µM acivicine for up to 90 min at 37 °C without or with 100 µM menadione in the absence or the presence of ritonavir or MK571 in the concentrations indicated. After the incubation, the incubation medium was harvested for quantification of the contents of extracellular GSx, GSSG and the extracellular LDH activity. The cells were washed with 1 mL of ice-cold PBS before the cells were lysed to determine the cellular contents of GSx and GSSG.

Cultured astrocytes have been reported to rapidly form the bimane-GSH conjugate GS-B during incubation with monochlorobimane (MCB), which is exported via Mrp1 [37, 38]. To investigate a potential influence of ritonavir on the GS-B export, cultured astrocytes were washed twice with 1 mL prewarmed (37 °C) IB and incubated in 500 µL IB containing 100 µM acivicin and 10 µM MCB in the absence or presence of ritonavir or MK571 in the concentrations indicated. After the incubation, the medium was collected and the cells were washed twice with 1 mL ice-cold PBS prior to lysis for determination of the cellular GSx and GS-B contents.

### Determination of Cell Viability and Cellular Protein Content

The cytosolic enzyme lactate dehydrogenase (LDH) is released from cells upon impairment of membrane integrity. Therefore, extracellular LDH activity was monitored as marker for potential cell damage and loss in cell viability as previously described in detail [53, 57]. The protein content of the cultures was measured by the Lowry method [58] using BSA as standard protein.

### Determination of the Contents of GSx, GSSG and GS-B

The amounts of cellular and extracellular GSx and GSSG were quantified by a modification of the colorimetric Tietze method for the use of 96-well microtiter plates as described previously [53]. To quantify extracellular GSx or GSSG contents, 10 µL media samples were mixed with 10 µL 1% (w/v) SSA and this mixture was applied to the test. The cells were lysed with 200 µL 1% (w/v) SSA and 10 µL of these lysates were used for the quantification of the cellular GSx and GSSG contents.

The cellular and extracellular GS-B contents were determined as described previously [37, 38]. After the given incubation, the cells were lysed in 500 µL lysis buffer (1% (w/v) Triton X-100 in 20 mM potassium phosphate buffer, pH 6.5). 200 µL of the harvested incubation media or of cell lysates were transferred into wells of a black 96-well microtiter plate and the GS-B fluorescence was recorded at 520 nm after excitation at 390 nm using a Thermo Labsystems Fluoroskan Ascent FL fluorescence microtiter plate reader (Fisher Scientific, Schwerte, Germany). For GS-B quantification, the fluorescence of samples was compared to those of GS-B standards in incubation buffer or in lysis buffer, which had been prepared as described previously [37, 38].

### Atomistic Model of Rat Mrp1 and Amino Acid Sequence Alignment

Due to missing structural data for rat Mrp1, a homology model was built by the I-TASSER web server for protein structure and function predictions [59, 60]. In order to decrease the model size and improve the computational efficiency, only amino acids 194–1532 (UniProtKB-Q8CG09) were included into the structure generation process, disregarding the N-terminal transmembrane domain (TMD) 0. Truncation mutants for human and bovine Mrp1 devoid of these N-terminal residues did not show any functional alterations in transport and nucleotide-trapping assays, and no altered ATPase activity compared to the full-length construct [12, 61]. Therefore, the shortened protein model was assumed to be a valid starting structure to assess ligand binding. Templates employed in the threading algorithm were bovine Mrp1 (PDB entries: 5uj9, 6bhu), cystic fibrosis transmembrane conductance regulator from zebrafish (PDB entry: 5uar) and pancreatic ATP-sensitive K^+^ channel SUR1/Kir6.2 (PDB entry: 5twv) (Fig. S4a). The final model obtained a confidence score of -0.2, where a higher value signifies a higher confidence. A confidence score of above − 1.5 means that more than 90% of the predictions are correct and therefore our rat Mrp1 model is considered to be predicted with an overall correct fold [59]. A detailed homology model report, generated by I-TASSER, was uploaded to 10.5281/zenodo.6592231.

### Construction of the Model Systems for the MD Simulations

In order to create a simulation box containing the biomolecular models, and to setup all simulation parameters, the CHARMM-GUI Modeller [62] was employed. First, the atomistic model of rat Mrp1 was incorporated into a lipid bilayer with a composition mimicking the one of rat astrocytes. Due to the complexity of the actual astrocyte membrane composition, including many different phospholipids with varying fatty acid tails [63, 64], a simplified but still representative version was constructed. An average composition consisting of 50% phosphatidylcholines (PC), 34% phosphatidylethanolamines (PE) and 16% cholesterol was chosen. Different phospholipids were selected for each class, to achieve a heterogenous fatty acid composition as reported in experiments for choline and ethanolamine glycerophospholipids in rat astrocytes: 31% 1-palmitoyl-2-oleoylphosphocholine (POPC), 7% 1-palmitoyl-2-arachidonoylphosphatidylcholine (PAPC), 5% 1-stearoyl-2-oleoylphosphatidylcholine (SOPC), 5% 1,2-dioleoylphosphocholine (DOPC), 2% 1-stearoyl-2-docosahexaenoylphosphatidylcholine (SDPC), 8% 1-stearoyl-2-oleoylphosphatidyl-ethanolamine (SOPE), 7% 1-stearoyl-2-docosahexaenoyl-phosphatidylethanolamine (SDPE), 6% 1,2-dioleoylphosphoethanolamine (DOPE), 5% 1-palmitoyl-2-oleoylphosphatidyl-ethanolamine (POPE), 5% diacylglycerophosphatidylethanolamine (DAPE), 3% 1-stearoyl-2-arachidonoylphosphoethanolamine (SAPE) [63]. In each simulation one layer of the membrane was composed of 336 lipids and 64 cholesterol molecules. The bilayer was treated homogenously, having the same composition in the inner and outer leaflet. The membrane was positioned perpendicular to the z axis on all simulations, spanning the xy plane. Water molecules were used to fill the box, achieving a distance of 15 Å between the protein and box edges. 145 mM of sodium and chloride ions were placed for charge neutrality, representing the experimental conditions. The final box dimensions were around 16 × 16 × 17 nm^3^ with around 460,000 atoms. In case of protein-ligand simulations, the simulation box was built in the same way as described above. As starting structure, a complex in which the ligand had been docked to the binding site of rat Mrp1 derived from docking simulations was used.

### MD Simulations

MD simulations were performed with the GROMACS 2018 package [65] employing the CHARMM36m force field [66] for protein and ligand molecules, whereas the TIP3P model was used for water [67]. CHARMM36 was additionally used for lipid molecules, as it is known for its accurate reproduction of experimental lipid properties [68]. As an integration scheme the leap-frog algorithm was employed with a timestep of 2 fs. Constraints of bonds connected to hydrogen atoms were performed with the LINCS algorithm [69].

Minimization and equilibration routines were performed as suggested by the CHARMM-GUI membrane builder [70, 71] for transmembrane systems in seven consecutive steps. First a minimization using the steepest descent algorithm with a tolerance of 1000 kJ mol^-1^ nm^-1^ in combination with position and dihedral restraints on heavy atoms of Mrp1 and lipids was performed. Subsequently, two equilibration steps under the NVT ensemble for 250 ps each were followed by four NPT simulations with a total length of 1750 ps. Positional and dihedral restraints were gradually decreased over the different equilibration steps, to fully relax the system. Production runs were performed unrestrained for 1000 ns under the NPT ensemble, recording atomic positions every 100 ps. During equilibration, temperature coupling was achieved by the Berendsen thermostat with a tau of 1.0 ps and pressure coupling by a semiisotropic Berendsen barostat with a tau of 5.0 ps [72], treating the z axis, lying perpendicular to the membrane, differently from the x and y axes. The system’s temperature was kept at 303.15 K and the reference pressure set to 1 bar, with a compressibility of 4.5 × 10^− 5^ bar^− 1^ for all simulations. The Verlet cut-off scheme [73] was used for treating van der Waals parameters under the PME method and the standardized parameters suggested for CHARMM36 in the GROMACS manual version 2019.

### Ligand Molecules Parametrisation

Force-field parameters for GSH and GSSG were derived for the CHARMM36m force field. Due to their peptide-like character, most parameters could be derived from existing entries, namely from glutamic acid, cysteine and glycine. In detail, atom types and charges for the cysteine and glycine portion of GSH were directly used from the respective amino acids. However, the γ-glutamyl residue in GSH is connected via an isopeptide bond to cysteine through its side chain. Therefore, the pristine glutamic acid parameters were modified to make the side chain less electronegative and create a peptide-bond-like character, as suggested in the “Topology file tutorial” by the Theoretical and Computational Biophysics group [74]. Additionally, as the γ-glutamyl part resembles both a normal N-terminus and a normal C-terminus, and the glycine part a normal C-terminus, standard atom types and charges have been used. GSSG was constructed from two GSH molecules by modifying both cysteine residues to disulfide-forming cysteines. Here, the electronegativity of both sulfur atoms was reduced as recommended in the CHARMM36m force field. Simulation parameters for ritonavir were generated using the CHARMM-GUI ligand reader and modeler [62, 75], proposing topology and parameter files for the CHARMM General Forcefield (CgenFF) version 2.4.0 [76]. Force-field parameters in the form of itp files are available under 10.5281/zenodo.6592231 for all ligands.

### Protein-Ligand Docking

In order to generate protein-ligand complex configurations as starting structures for MD simulations, docking of the individual ligands GSH, GSSG and ritonavir was performed to the receptor rat Mrp1. Initial atomic coordinates of ligand conformations were generated with the CHARMM-GUI ligand reader and modeller. The starting structure of rat Mrp1 was derived after an unrestrained MD simulation of 50 ns, simulating the protein embedded in a membrane under physiological conditions. The equilibrated structure was in an apo-form, and the membrane and solvent molecules were removed for the subsequent docking procedure. AutoDock version 4.2.6 was used in this study, employing the Lamarckian genetic algorithm with 50 dockings executed for each ligand [77, 78]. AutoDockTools were used for coordinate preparation [79] of protein and ligand molecules, namely merging nonpolar hydrogens and adding charges. The protein was kept rigid during docking, whereas GSH, GSSG and ritonavir had 11, 23, and 19 rotatable bonds defined, respectively. Non-rotatable bonds were peptide bonds, double bonds and any bond to a hydrogen. The binding site was defined around the P- and H-pocket of Mrp1 with 82,78,60 grid points in x,y,z. The sampling grid was 0.375 Å. Docking parameter files as well as the pdbqt files, containing all 50 docking poses for each ligand, are stored at 10.5281/zenodo.6592231.

### RMSD and Hydrogen Bond Calculation

The root-mean square deviation (RMSD) of GSH over the trajectory was computed using the open-source, community-developed PLUMED library version 2.6 [80, 81] together with the Python package MDAnalysis [82, 83]. In order to monitor the movement of the ligand with respect to the binding site of the protein, an alignment of frames was done on the heavy atoms of the protein backbone and only displacement calculations on the atomic position of the ligand molecule were performed. The optimal alignment method calculated using the Kearsley algorithm was employed [84], in which the displacement of GSH was calculated after the alignment of geometric centers of the instantaneous and reference configuration as well as alignment of both frames. Hydrogen bonds have been calculated using the VMD plugin Hbonds. A cut-off distance of 3 Å has been chosen between donor and receptor, together with a cut-off angle of 20 degrees. Hydrogen bonds were evaluated every 1 ns.

### Presentation of Data

All experimental quantitative data shown represent means ± standard deviation (SD) of values that were obtained in experiments performed on at least three independently prepared astrocyte cultures. The significance of differences between multiple groups of data was analysed by ANOVA followed by the Bonferroni *post-hoc* test. The t-test was used for statistical comparison of two sets of data. p > 0.05 was considered as not significant.

### Data Availability

Molecular docking and MD simulations performed in this study can be further analyzed or reproduced by their associated files, namely structures, trajectories, movies and forcefield parameter files for ligands, made available under 10.5281/zenodo.6592231. PLUMED input files required to reproduce results reported in this paper are available on PLUMED-NEST (www.plumed-nest.org), the public repository of the PLUMED consortium, as plumID:22.022.

## Results

### Test for Cell Viability

For each experiment performed and presented in this study, the viability of the cells was investigated by quantification of the extracellular activity of lactate dehydrogenase (LDH). For none of the experimental conditions used, a significant increase in the extracellular LDH activity was observed compared to control (Fig. S1-S3). Thus, a potential cyto-toxicity of the incubation conditions can be excluded as reason for a release of the three Mrp1 substrates GSH, GSSG and GS-B from the cells into the media. These results confirm the reported low toxic potential of ritonavir, MK571, menadione and MCB in the applied concentrations for short-term incubations within the hour range [18, 37, 38, 52, 56].

### Test for a Potential Influence of Ritonavir on the Time-Dependent Release of GSH from Cultured Astrocytes

**Fig. 1:**
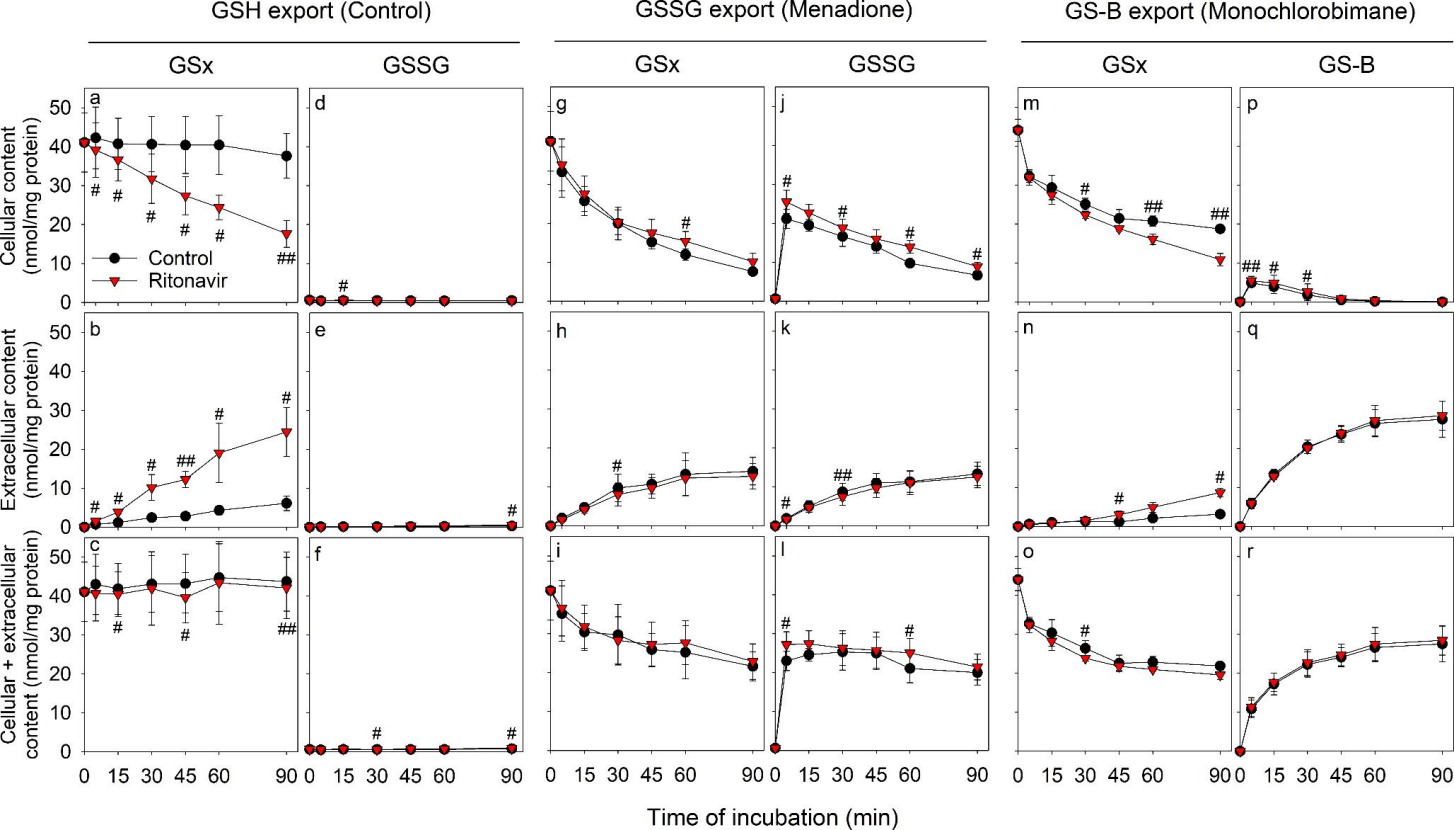
Time-dependent effects of ritonavir on the export of the Mrp1 substrates GSH, GSSG and GS-B from cultured astrocytes. The cells were incubated for up to 90 min without or with 10 µM ritonavir in the absence **(a-f)** or presence of 100 µM menadione **(g-l)** or 10 µM MCB **(m-r)**. Afterwards, the cellular, extracellular and the sum of cellular plus extracellular contents of GSx (a-c; g-i; m-o), GSSG (d-f; j-l) or GS-B (p-r) were determined. The initial specific cellular GSx contents of the cultures were 41 ± 8 nmol/mg (a-l) and 44 ± 3 nmol/mg (m-r) and the cultures contained 149 ± 8 (a-l) and 157 ± 20 (m-r) µg protein per well. The data shown represent means ± SD of values that had been obtained in experiments performed on three independently prepared astrocyte cultures. Statistical analysis of the significance of differences of the data obtained after incubation without (control) and with ritonavir was performed by one-tailed Student’s t-test. The levels of significance are indicated by ^#^p < 0.05, ^##^p < 0.01 and ^###^p < 0.001

In the absence of ritonavir, astrocyte cultures released only small amounts of GSH into the incubation medium during 90 min of incubation, as demonstrated by the nearly unaltered cellular GSx content (Fig. 1a) and the low time-dependent increase in the extracellular GSx content (Fig. 1b), whereas the sum of cellular plus extracellular GSx contents remained unchanged throughout the incubation period (Fig. 1c). In contrast, the presence of 10 µM ritonavir drastically increased the release of GSH from primary astrocyte cultures, confirming previous studies [52]. In comparison to control cells, the GSx contents of cells that had been incubated with ritonavir were significantly lower and decreased in a time-dependent manner (Fig. 1a). This was accompanied by a significant time-dependent increase in the extracellular GSx content (Fig. 1b), while the sum of cellular plus extracellular GSx contents remained constant throughout 90 min of incubation (Fig. 1c). After 90 min of incubation, about 60% of the cellular GSH had been exported in presence of ritonavir, whereas only around 10% had been exported from control cells (absence of ritonavir) (Fig. 1a,b).

Astrocyte cultures contained very low amounts (below 0.6 nmol/mg protein) of GSSG after incubation without or with ritonavir (Fig. 1d-f). Thus, the observed stimulation of extracellular GSH accumulation by ritonavir was considered to be exclusively due to GSH export.

### Test for a Potential Influence of Ritonavir on the Time-Dependent Release of GSSG from Astrocytes after Menadione-Induced GSH Oxidation

Unstressed cultured astrocytes contain hardly any GSSG. Thus, to test for a potential effect of ritonavir on the export of GSSG, astrocytes were exposed to oxidative stress by application of 100 µM menadione as stressor, as recently described [37], in the absence or the presence of ritonavir (Fig. 1g-l), which caused a rapid oxidation of cellular GSH to GSSG already within 5 min of incubation (Fig. 1j). The formed GSSG was continuously exported from the cells as shown by the time-dependent decrease in cellular GSx and GSSG (Fig. 1g, j), which was accompanied by a matching increase in the extracellular GSx and GSSG contents (Fig. 1h, k), whereas the sum of cellular plus extracellular GSSG remained almost constant (Fig. 1l). The total amounts of GSx (cellular plus extracellular values) decreased in the beginning of the incubation (Fig. 1i) but were almost identical to the total GSSG content towards the end of the incubation time (Fig. 1l). Likely reasons for the disappearance of some GSx from menadione-treated cultures are the conjugation between GSH and menadione [37, 56, 85] and/or the formation of GSH-protein mixed disulfides, which has been reported for cells that contain high GSSG contents [86, 87].

The presence of ritonavir in a concentration of 10 µM had at best only a minor influence on the release of GSSG from astrocytes as indicated by the slightly higher cellular GSSG contents in cells exposed to 10 µM ritonavir (Fig. 1l), while extracellular and total GSSG values were almost identical for control and ritonavir-treated cultures (Fig. 1k, l). This clearly demonstrates that ritonavir does not stimulate GSSG export from astrocytes.

### Test for a Potential Influence of Ritonavir on the Time-Dependent Release of GS-B

To test for a potential influence of ritonavir on the export of GS-B, cultured astrocytes were incubated with 10 µM monochlorobimane (MCB) in the absence or presence of 10 µM ritonavir for up to 90 min to generate cellular GS-B, as recently described [37] (Fig. 1m-r). Already after 5 min of incubation, a maximal cellular GS-B content of around 5 nmol/mg protein was determined, which decreased during further incubation until GS-B was no longer detectable anymore in the cells after 60 min of incubation (Fig. 1p). In contrast, the extracellular and total (sum of cellular plus extracellular) GS-B content increased strongly during the initial part of the incubation and reached maximal values of around 28 nmol/mg after incubation for around 60 min (Fig. 1q, r). Accordingly, the cellular GSx content decreased during 60 min incubation to a value of around 22 nmol/mg (Fig. 1m).

Within 45 min of incubation, all MCB applied (10 µM = 2 nmol/well) seemed to have reacted to GS-B, while during longer incubations, export of the remaining GSH from the cells was observed, which was stimulated in the presence of ritonavir (Fig. 1m,n). In contrast, the export of GS-B from astrocytes was not stimulated by the presence of 10 µM ritonavir (Fig. 1q). In fact, compared to control cells, a slightly but significantly elevated cellular GS-B content was determined for cells that had been incubated with MCB in the presence of ritonavir (Fig. 1p).

### Impact of Ritonavir on the Kinetic Parameters of the GSH Export from Astrocytes

**Fig. 2:**
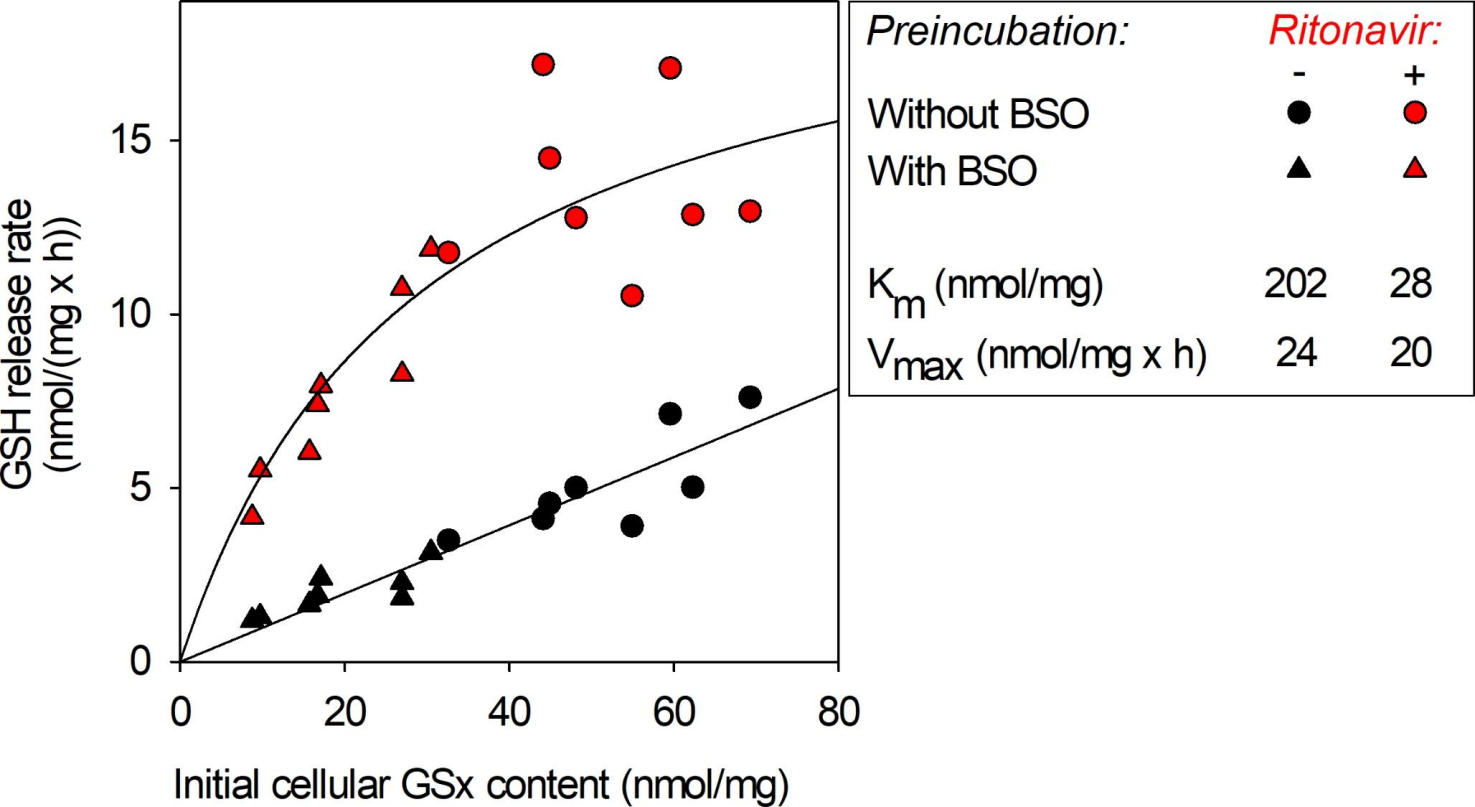
Kinetic analysis of the modification by ritonavir on the GSH release rate from primary astrocyte cultures. The cells were preincubated without (circles) or with 100 µM BSO (triangles) for up to 24 h to obtain diverse initial specific cellular GSx contents. Afterwards, the cells were incubated in the absence of BSO without (black symbols) or with 10 µM ritonavir (red symbols) for up to 3 h. The initial linear increase in the extracellular GSx contents between 10 and 60 min (ritonavir-treatment) and between 30 and 180 min (no ritonavir-treatment) were used to calculate the specific GSH release rates, which were plotted against the specific initial cellular GSx content. The values for K_m_ and V_max_ were calculated from the data shown in the graph by using the Hanes-Woolf linearization

To investigate the mechanism involved in the ritonavir-stimulated GSH export from astrocytes, the kinetic parameters of the GSH export were investigated for control and ritonavir-treated cultures. To obtain cultured astrocytes that contain different cellular GSH concentrations, the cells were pre-incubated in the absence or the presence of the GSH synthesis inhibitor L-buthionine sulfoximine (BSO) [88, 89] for time intervals of up to 24 h. These pre-incubations established specific cellular GSx contents ranging between 10 and 70 nmol/mg (Fig. 2). Determination of the initial GSH export rates of those pretreated astrocytes revealed that the basal GSH release rate was nearly proportional to the initial cellular GSx content in astrocytes which had been incubated in the absence of ritonavir (Fig. 2), as reported previously [88–90]. In contrast, the GSH export was strongly accelerated in ritonavir-treated astrocytes for all cellular GSx contents investigated and the GSH release rate showed a hyperbolic dependency on the initial cellular GSx content (Fig. 2).

Hyperbolic fittings for the dependency of the GSH release rates on the initial cellular GSx contents by the Michaelis-Menten equation resulted in reasonably high correlation coefficients of 0.87 (control cells) and 0.76 (ritonavir-treated cells) (Fig. 2). The data shown in Fig. 2 were linearised by using the Hanes-Woolf plot to calculate K_m_ and V_max_ values (Fig. 2). For astrocytes incubated without ritonavir, the export of GSH from these cells was found to have a K_m_ value of 202 nmol/mg and a V_max_ value of 24 nmol/(mg x h) (Fig. 2). In contrast, for ritonavir-treated cells, the K_m_ value for the export of GSH was lowered by a factor of more than 7, and amounted to 28 nmol/mg, while the V_max_ value determined for those cells remained with 20 nmol/(mg x h) quite similar to that calculated for control cells.

### Inhibition of Mrp1-mediated Transport of GSSG and GS-B by Higher Concentrations of Ritonavir

**Fig. 3:**
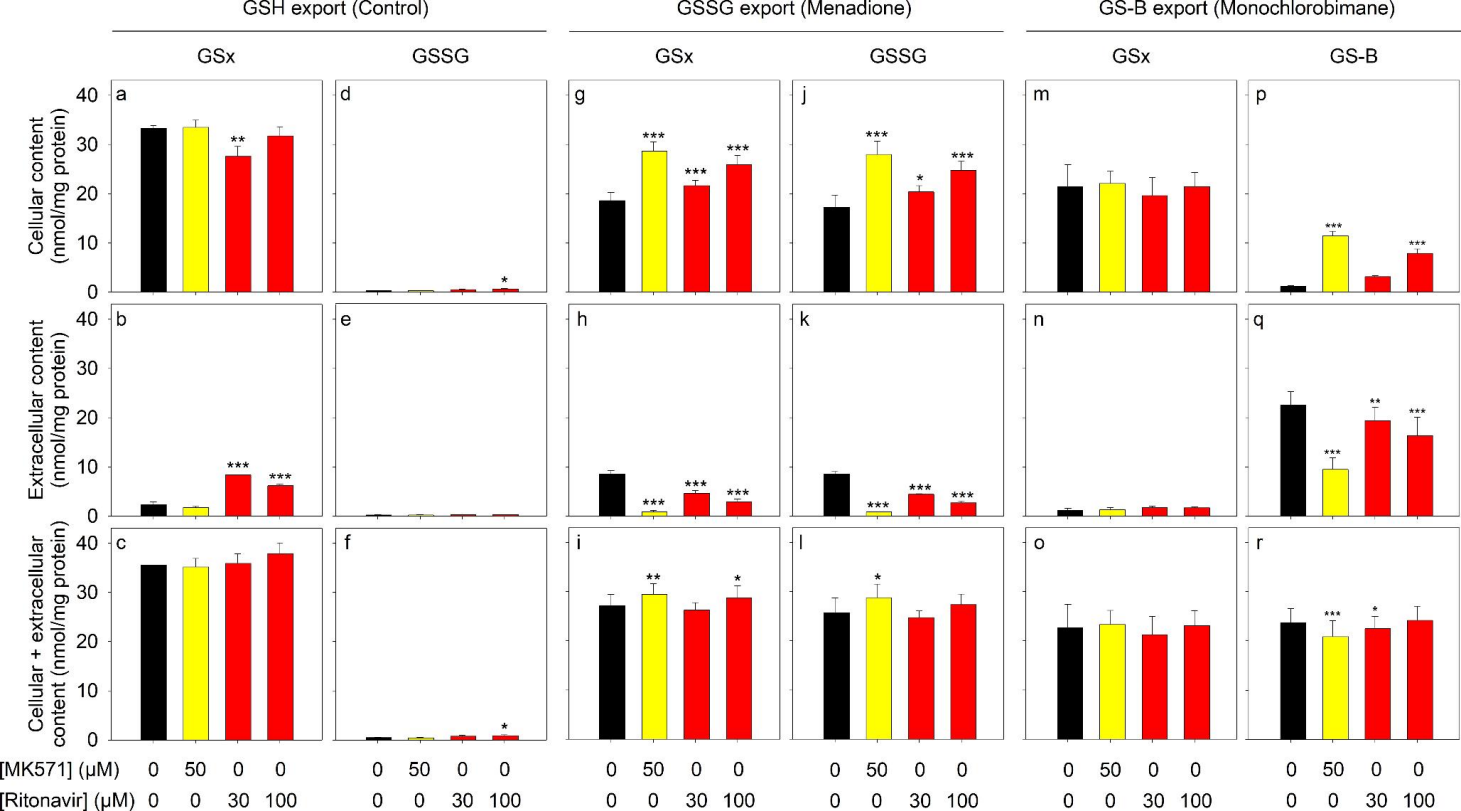
Concentration-dependent effects of ritonavir on the export of the Mrp1 substrates GSH, GSSG and GS-B from cultured astrocytes. The cells were incubated for 30 min without or with 50 µM MK571, 30 µM ritonavir or 100 µM ritonavir in the absence **(a-f)** or the presence of 100 µM menadione **(g-l)** or 10 µM MCB **(m-r)**. Afterwards, the cellular, extracellular and the sum of cellular plus extracellular contents of GSx (a-c; g-i; m-o), GSSG (d-f; j-l) or GS-B (p-r) were determined. The specific initial cellular GSx contents of the cultures were 33.7 ± 0.1 nmol/mg (a-l) and 41.1 ± 0.1 nmol/mg (m-r) and the cultures contained 147 ± 24 (a-l) and 154 ± 6 (m-r) µg protein per well. The data shown represent mean ± SD of values that had been obtained in experiments performed on three independently prepared astrocyte cultures. Significant differences of the data obtained after incubation without (control) compared to incubation with ritonavir or MK571 were analysed with ANOVA followed by the Bonferroni post hoc-test. The levels of significance are indicated by *p < 0.05, **p < 0.01 and ***p < 0.001

Ritonavir in a concentration of 10 µM stimulated GSH export, but not the export of GSSG and GS-B from astrocytes (Fig. 1). In order to test for the potential of ritonavir to affect Mrp1-mediated export processes from astrocytes in higher concentrations than 10 µM, ritonavir was applied in concentrations of 30 µM or 100 µM for 30 min, before the export of Mrp1 substrates was studied, as described above. Compared to the respective control incubations, 30 µM or 100 µM ritonavir stimulated the export of GSH from viable astrocytes (Fig. 3a-f). In contrast, the export of GSSG from menadione-treated astrocytes (Fig. 3g-l) as well as the export of GS-B from MCB-treated astrocytes (Fig. 3m-r) was significantly lowered compared to the respective control incubations (absence of ritonavir). Incubations with MK571 as positive control for the inhibition of Mrp1-mediated transport processes [16–18, 38] confirmed that the export of GSSG and GS-B observed under the conditions used was significantly lowered in the presence of this known Mrp1 inhibitor (Fig. 3).

### Sequence Comparison and Structure Prediction of Rat Mrp1

**Fig. 4:**
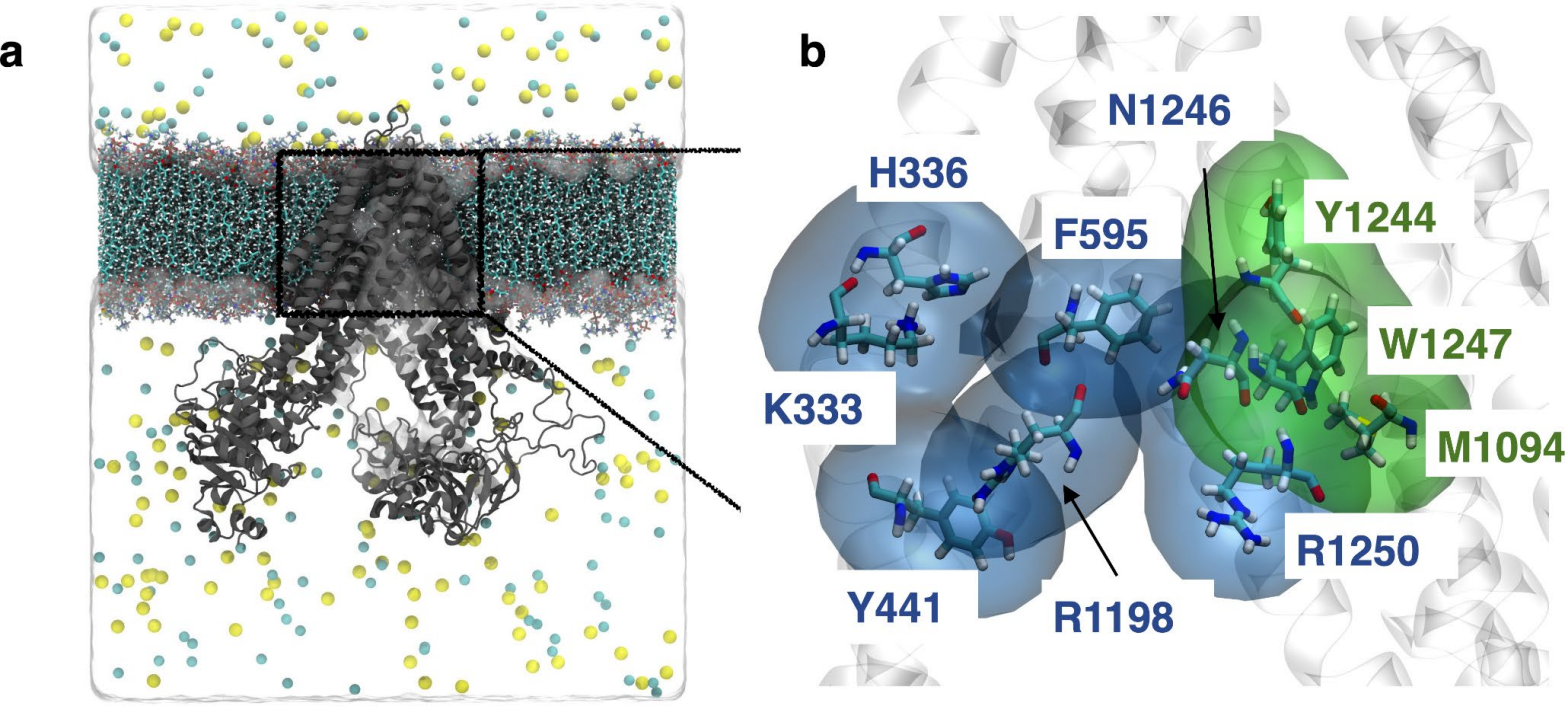
Homology model of rat Mrp1. Atomistic structure of the rat Mrp1 homology model (cartoon style in grey), embedded in an astrocyte-like cell membrane (licorice) and surrounded by water (not shown) and ion molecules (sodium = yellow, chloride = cyan) **(a)**. Closeup of the binding site of rat Mrp1 with important amino acids represented as licorice models. Volume surfaces of the P-pocket and H-pocket are represented in transparent blue and green, respectively **(b)**. Lipids and amino acids are colour-labelled by their atom composition with carbon = cyan, nitrogen = blue, oxygen = red, hydrogen = white

Ritonavir was shown to stimulate the Mrp1-mediated GSH export (Figs. 1a and b and 2) and to inhibit the Mrp1-mediated GSSG (Fig. 3j,k) and GS-B (Fig. 3p,q) export from viable rat astrocytes. In order to investigate whether the binding of ritonavir to Mrp1 interferes with the binding of physiological substrates such as GSH or GSSG, an *in silico* approach employing ligand docking and subsequent MD simulations was employed. The structure of rat Mrp1 has not been reported so far in contrast to that of bovine Mrp1, resolved via cryo-electron microscopy [12, 13]. The domain structures of bovine, human and rat Mrp1 are highly similar and the amino acid sequence alignments of Mrp1 from all three species revealed around 90% sequence identity (data not shown). Hence, a homology model for rat Mrp1 was constructed by means of the I-TASSER server [91–93] and subsequently positioned in a lipid bilayer composed of an astrocyte-specific composition of membrane lipids (Fig. 4a). Structural features were mostly derived from the following templates: bovine Mrp1 (pdb entries: 5UJ9 and 6BHU), cystic fibrosis transmembrane conductance regulator (CFTR) from zebrafish (pdb entry: 5UAR), potassium channel from rat (pdb entry: 5TWV) (Fig. S4a). Evaluation of the homology modelling scores is given in more detail in the materials and methods section. The generated rat Mrp1 model showed an overall similar topology compared to bovine Mrp1 and structurally aligned regions harbour a root-mean-square deviation (RMSD) of 0.71 Å (Fig. S4b). Amino acids predicted to be involved in binding are conserved both sequence-wise and structure-wise between rat and bovine Mrp1 (Fig. S4c,d).

### Simultaneous Binding of Ritonavir and GSH to Rat Mrp1

As proposed by Hirrlinger *et al.* [17] for rat Mrp1 and confirmed for bovine Mrp1 by Johnson and Chen [12], the ligand-binding pocket of Mrp1 contains a positive hydrophilic (P) and a hydrophobic (H) part (Fig. 4b). The position of the GSH-conjugate LTC4 was examined for bovine Mrp1 via cryo-EM, identifying its GS-moiety binding to the P-pocket via hydrogen bonds with both of its carboxyl groups [12]. However, the binding preferences of the endogenous substrates GSH and GSSG have not been resolved so far. Additionally, ritonavir binding to Mrp1’s binding pocket has not been reported before and was only postulated due to experimental findings [52]. In order to investigate potential binding and binding preferences of these substrates to the binding pocket of Mrp1, initial protein-ligand complex structures as starting point for later MD simulations were obtained by a molecular docking analysis, which was performed for GSH, GSSG or ritonavir into the binding site of rat Mrp1. Results of these ligand docking experiments are described and interpreted in detail in the Supporting Information (Fig S5). Subsequent MD simulations were performed to investigate the probable influence of ritonavir on the binding of GSH or GSSG over time on an atomistic scale.

**Fig. 5:**
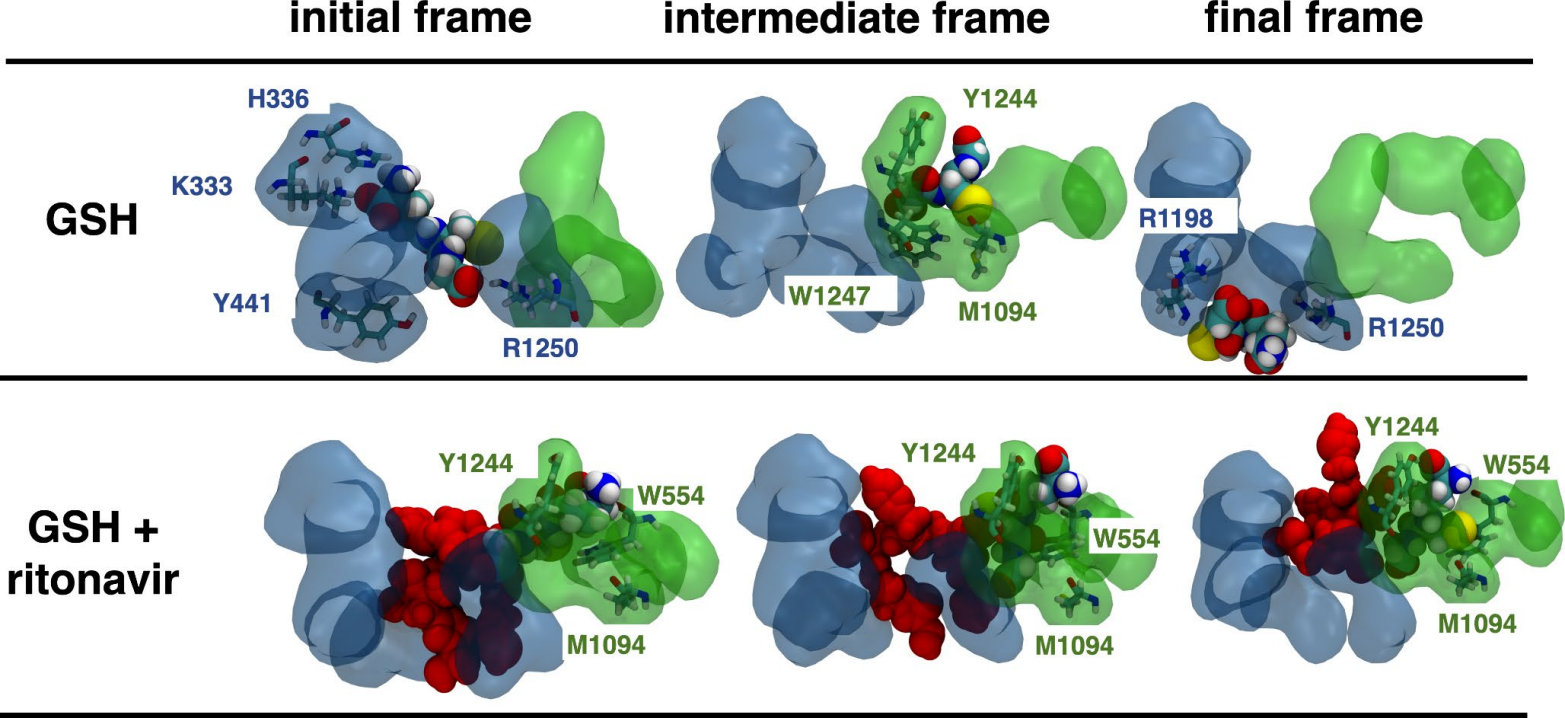
Representative ligand binding poses of GSH in absence or presence of ritonavir within the binding pocket of rat Mrp1 observed in MD simulations of 1 µs. The initial frame, an intermediate frame with key interactions and the final frame of each simulation is shown for all ligand and ligand combinations. GSH binding mode to the P-pocket of rat Mrp1, resembling the experimentally determined binding pose of the GS-moiety of LTC4 to bovine Mrp1 [12]. Binding poses of GSH to the P-pocket before diffusing into the H-pocket, binding there for around 800 ns. Simultaneous binding of GSH and ritonavir (red) resulted in a stable complex formation that remained stable over the whole simulation time. Volume surfaces of the P-pocket and H-pocket are represented in a transparent blue and green, respectively. GSH and amino acids are colour-labelled by their atom composition with carbon = cyan, nitrogen = blue, oxygen = red, hydrogen = white. Ritonavir molecules in combination with GSH are labelled in red. Only amino acids close to the ligand where explicitly shown. Lowest binding free energy structures were selected as starting formations from the ligand docking experiments. Movies of the trajectory progression can also be found under 10.5281/zenodo.6592231 for all conditions investigated

In a first step, MD simulations of 1 µs were performed for the individual substrates starting from a bound state in the rat Mrp1 binding site. Due to similar predicted binding affinities of GSH for the P-pocket and the H-pocket after docking, MD simulations with GSH were initially started from the P-pocket, in line with the experimentally determined position of the GSH-moiety of LTC4 [12]. Detailed analysis of binding configuration of GSH to the amino acids of the P-pocket revealed in fact the same binding mode as reported for the GS-moiety of LTC4. In detail, the carboxyl group of glycine in GSH formed hydrogen bonds to R1250, whereas the carboxyl group of the γ-glutamyl moiety experienced contacts with K333 and H336 (Figs. 5 and S5b). The agreement of binding modes between simulation and experiments underlines the ability of the constructed model to support experimental findings.

Over the simulation time of 1 µs, GSH showed an ambiguous behaviour, as it was able to move away from its starting position within the P-pocket and migrate into the H-pocket, spending the same amount of simulation time in each of the two (Fig. 5). In contrast, when GSSG was initially docked into the P-pocket, it left its position already after 150 ns to interact with amino acid and lipid residues underneath the binding site (to the direction of the cytosol), before diffusing off into the solvent after 460 ns (Fig. 5). The main interactions between GSSG and the binding site of Mrp1 were similar to those observed for GSH (hydrogen bonds to K333, Y441, R1198 and R1250) (Fig. S5c).

MD simulations with ritonavir showed that it remained for 750 ns in the P-pocket, before diffusing into the direction of the H-pocket (Figs. S5d and S6). However, ritonavir did not bind to the H-pocket but rather settled in close proximity, as already observed in our docking analysis.

In a second step, MD simulations were performed with either GSH and ritonavir or GSSG and ritonavir together within the binding site of rat Mrp1. For the simulation of GSH and ritonavir, GSH was initially placed into the H-pocket, whereas ritonavir was initially placed into the P-pocket, in agreement with the predicted docking free energy preferences (Fig. S5a). Over a simulation time of 1 µs, both ligand molecules remained in their own respective pockets, with little rearrangement of functional groups (Fig. 5). In contrast, when GSSG was initially placed in the P-pocket and ritonavir below the H-pocket (as predicted beforehand), over the simulation time of 1 µs ritonavir remained close to its initial binding location facing the cytosol, whereas GSSG left the P-pocket after 340 ns towards the cytosol and settled in close contact to ritonavir below the binding site (Fig S6).

### Stabilizing Effect of Ritonavir on GSH Binding to Rat Mrp1

**Fig. 6:**
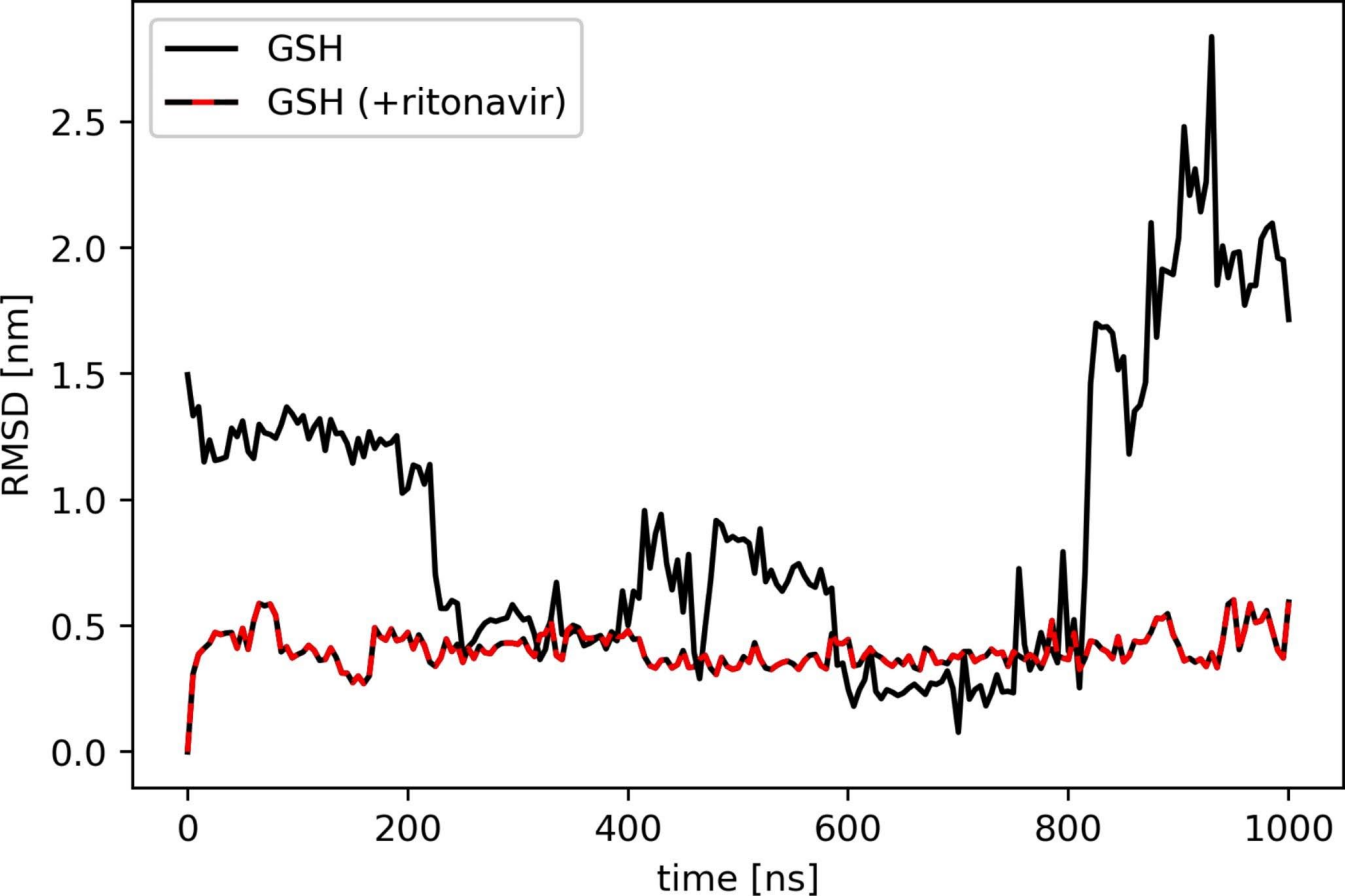
Stabilizing effect of ritonavir on the binding of GSH to rat Mrp1. RMSD [nm] of GSH computed from MD simulations of GSH binding alone or simultaneously with ritonavir to the binding site of rat Mrp1. Structures of GSH positioned in the H-pocket were used as reference structures for computation

To investigate the observed stimulation of GSH export in presence of the HIV protease inhibitor ritonavir, the influence of ritonavir on GSH binding to rat Mrp1 was further quantified by calculation of the RMSD of GSH during MD simulations in absence or presence of ritonavir. Tracking the movement of GSH in relation to the underlying protein structure was made possible by aligning the protein backbone of rat Mrp1 to a reference structure, in which GSH was bound to the H-pocket, prior to the calculation of GSH displacement (Fig. 6). When only GSH was bound to Mrp1, the RMSD decreased from 1.5 nm to 0.5 nm after 200 ns of simulation, which represents the diffusion of GSH from the P-pocket into the H-pocket, before leaving the binding site after 800 ns. Interestingly, such type of fluctuations were not observed during the simulation of GSH and ritonavir both bound to Mrp1, as GSH remained stably within the H-pocket throughout the whole simulation time (Fig. 6). The RMSD value in this case was predominantly constant and only slightly fluctuating around a value of 0.5 nm. Evaluation of interactions between GSH, in complex with ritonavir, and proximate amino acid side chains demonstrated persistent hydrogen bond formation to four amino acid residues comprising the binding pocket of rat Mrp1 (Fig S7). Three out of these (W554, Y1244, and R1250) had already been identified and reported as characteristic amino acids involved in ligand binding by Mrp1 within the H-pocket, and the P-pocket, respectively [12]. However, to our knowledge, the fourth amino acid (R594) has not yet been described to be involved in ligand-binding of Mrp1. R594 is part of to the P-pocket due to its positive partial charge, but is spatially close to the H-pocket. Despite the overall hydrophobic and less positively charged characteristics of the H-pocket compared to the P-pocket, GSH was still able to form hydrogen bonds to polar residues therein.

## Discussion

Cultured rat astrocytes were used to investigate the mechanisms involved in the modulation of the Mrp1-mediated export of GSH, GSSG and GS-B by the antiviral compound ritonavir. The release of GSH from viable astrocytes was strongly stimulated in the presence of 10 µM ritonavir as indicated by a 4-fold increased specific release rate, which is consistent with literature data [52]. Also formaldehyde has previously been reported to stimulate Mrp1-mediated GSH export from cultured astrocytes and this stimulation was characterised by a strong increase in the V_max_ value for the export of GSH, while the K_m_ values remained almost unchanged [88]. For this formaldehyde-induced stimulation, an increase in the number of active Mrp1 molecules in the cell membrane was postulated [88]. An increased number of Mrp1 proteins in the cell membrane could in principle also explain the ritonavir-induced stimulation of GSH export observed in this study. At least for ritonavir-treated human intestinal carcinoma cells, an upregulation of Mrp1 expression and activity after chronic exposure to ritonavir has been reported [94]. However, this appears unlikely to be the case for ritonavir-treated astrocytes, due to the short incubation times in the amino acid-free incubation medium that has been used. A recruitment of intracellular Mrp1-containing vesicles to the plasma membrane upon incubation with ritonavir, as reported for bilirubin-treated astrocytes [95], cannot contribute to the stimulated GSH export either, because of three reasons: (1) The V_max_ values calculated for the GSH export remained very similar for control and ritonavir-treated astrocytes; (2) the stimulation was only observed for GSH and not for the other Mrp1 substrates GSSG and GS-B; and (3) no obvious difference was observed for the cellular localization of Mrp1 by immunocytochemical staining of control and ritonavir-treated astrocytes (Fig. S8).

Kinetic analysis of the export of GSH from viable astrocytes revealed a K_m_ value of around 200 nmol/mg, which corresponds to around 50 mM GSH considering the reported cytosolic volume of 4.1 µL/mg protein for cultured astrocytes [96]. This is similar to values previously reported for the release of GSH from cultured astrocytes [88, 89]. However, in the presence of ritonavir the K_m_ value for GSH export was drastically lowered by 85% to around 28 nmol/mg (corresponding to 6.7 mM GSH). Thus, half-maximal transport velocity is established in ritonavir-treated astrocytes at much lower GSH concentrations. The cytosolic GSH concentration in cultured astrocytes has been reported to be 8 mM [96]. Using the calculated kinetic parameters for GSH export from control and ritonavir-treated astrocytes to calculate the GSH export velocity for the cytosolic concentration of 8 mM GSH, we obtained initial export rates of 3.3 nmol/(mg x h) (control) and 10.9 nmol/(mg x h) (ritonavir). These values account for 14% and 54% of the calculated V_max_, respectively, and match with the observed almost 4-fold stimulation of GSH export in ritonavir-treated astrocytes.

In contrast to the astrocytic GSH export, the Mrp1-mediated export of GSSG and GS-B, [16, 18, 37, 38, 56, 97] was not stimulated in the presence of 10 µM ritonavir. A high concentration of 100 µM ritonavir even inhibited these Mrp1-mediated export processes. Similarly, also the known Mrp1 inhibitor MK571 has been observed to lower the astrocytic GSSG and GS-B export [16, 37, 38] and competition of MK571 with these substrates has been proposed as the reason for the observed inhibition [17]. A potentially stronger inhibitory effect of ritonavir in concentrations above 100 µM on the GSSG and GS-B export could unfortunately not be investigated due to the limited solubility of ritonavir. For peripheral cells, it was shown that ritonavir is itself a substrate of Mrp1 and thereby inhibits the transport of other Mrp1 substrates such as the chemotherapeutic drugs doxorubicin and etoposide [98–101]. The ritonavir-dependent inhibition of Mrp1-mediated export of GSSG and GS-B from astrocytes is consistent with these literature data. Thus, ritonavir differently modulates the transport properties of different Mrp1 substrates; it namely stimulates GSH export but inhibits GSSG and GS-B export from rat astrocytes.

**Fig. 7:**
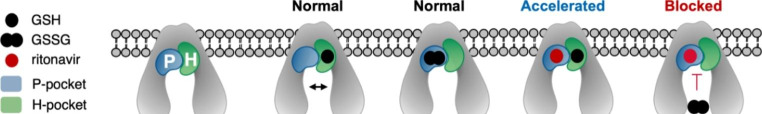
Hypothetical model explaining the observed influence of ritonavir on the export of GSH, GSSG and GS-B via Mrp1. Mrp1 contains a bipartite ligand binding site, that is composed of a polar part (P-pocket) and a hydrophobic part (H-pocket). GSH binds with high affinity to both sites, whereas bulkier compounds like GSSG and ritonavir preferentially occupy the P-pocket. In the absence of ritonavir, GSH might bind into one of either binding sites and is exported with normal velocity. When ritonavir occupies the P-site, a simultaneous binding of GSH to the H-pocket is not only still possible, but even stabilized, which accelerates its export. This happens as long as the concentration of ritonavir is not as high as to hinder the GSH entrance into the binding site. In contrast, GSSG and GS-B compete for the same P-pocket as ritonavir, which thus blocks the export of GSSG and GS-B in presence of ritonavir

To shed light onto this surprising finding, atomistic details of the above-mentioned diverse transport processes were investigated by MD simulations using the homology model of rat Mrp1 in complex with its docked ligands GSH, GSSG and/or ritonavir. The predicted binding position of GSH in the P-pocket of rat Mrp1 is similar to those found by Johnson and Chen [12] for the GS-moiety of LTC4 in bovine Mrp1 resolved by cryo-EM experiments, including hydrogen bond formation to residues R1198 and R1250. Therefore, the generated rat homology model was considered a useful tool for further rat Mrp1-ligand interaction studies. In the absence of other ligands, GSH was previously suggested to bind preferentially to the P-Pocket of Mrp1 [12]. In our simulations we have observed also a high affinity of GSH for the H-pocket (Figs. 5 and 7), which is likely caused by hydrogen-bond interactions to the amino acids Y1244, R1250, W554 and R594 (Fig. S7). Although R1250 and R594 actually belong to the P-pocket due to their positive charges, their spatial proximity to residues of the H-pocket enables the stabilization of GSH within the cavity of the H-pocket. In addition, residues W554 and R594 belong to the transmembrane domain (TMD) 1 bundle, whereas Y1244 and R1250 are classified within TMD2. Simultaneous binding of GSH to R1198, K333, H336, Y441 and R1250 (GSH binding to the P-pocket) or W554, R594, Y1244 and R1250 (GSH binding to H-pocket) would bridge TMD1 and TMD2 in both cases. This mechanism was suggested to initiate an overall conformational change in Mrp1, which is crucial for the transport process and also applies to the predicted GSH binding configurations in this study [12]. Due to the discovery of two translocation pathways, one from each pocket, which merge into the extracellular facing site of the transmembrane domain of Mrp1, transport of GSH from either pockets is theoretically possible [102].

The observed binding mode of GSSG to the P-pocket of rat Mrp1 resembled that of GSH within the P-pocket. However, the binding was much less stable, and GSSG was observed to migrate out of the pocket to diffuse away into the cytosol after a few hundred ns of simulation time (Fig. S6). Additionally, no binding of GSSG to the H-pocket could be identified.

Moreover, ritonavir preferentially binds to the P-pocket (Figs. S6 and 7). Binding to the H-pocket might be hindered due to ritonavir’s relatively large size (98 atoms). Indeed, the H-pocket only consists of five amino acids, whereas the P-pocket consists of up to ten amino acids, including R594 (Fig. S4c). The same explanation likely applies to GSSG (70 atoms), whereas the much smaller GSH molecule (36 atoms) readily binds to both the P-pocket and the H-pocket, as highlighted above.

As a consequence, the simultaneous binding of GSH and ritonavir to rat Mrp1 is sterically possible (Figs. 5 and 7). Additionally, the residence time and binding stability of GSH to rat Mrp1 was increased in presence of ritonavir (Fig. 6). The binding of ritonavir to Mrp1 may therefore increase the affinity of GSH for the H-Pocket of Mrp1, thereby enhancing GSH binding and lowering the K_m_ value for GSH transport. Seemingly, ritonavir acts like a plug when bound to the P-pocket, locking a GSH molecule in its position within the H-pocket until the transport process is initiated. This concept is in agreement with the recently reported hypothesis of sequential binding of GSH and modulators of Mrp1-mediated GSH export [14]. Amino acids W554, M1094, W1247 and R1250 were observed to spatially restrict the movement of GSH into the direction of the cytosol, while ritonavir blocks the movement towards the P-pocket, thereby preventing the diffusion of GSH out of the binding site. Application of ritonavir in higher concentrations (100 µM) to astrocyte cultures resulted in a decreased acceleration of Mrp1-mediated GSH export compared to applications of lower concentrations (30 µM) (Fig. 3a,b). This experimental finding could be explained by the predicted binding model discussed above. If ritonavir binds to the P-pocket before a GSH molecule is able to bind to the H-pocket, access of GSH to the entire binding site might be blocked by ritonavir and the transport process be brought to a halt for the duration of ritonavir binding.

In contrast, ritonavir is likely to compete with GSSG for binding at the same P-pocket of the Mrp1 binding site, thereby inhibiting the binding and the subsequent transport of this Mrp1 substrate (Figs. S6 and 7). An inhibitory mechanism on the GSSG export was already postulated for MK571. However, it was suggested that MK571 would bind to the H-pocket [17]. The compound GS-B (61 atoms) is structurally similar to GSSG and its export was indeed reduced upon addition of ritonavir. Although GS-B was not simulated explicitly, it is plausible that it also preferentially occupies the P-pocket, thus competes for binding with ritonavir.

In conclusion, the experimentally observed interference of the antiretroviral drug ritonavir with the export of the different Mrp1 substrates GSH, GSSG and GS-B can most likely be explained by direct binding of ritonavir into the polar part of the biphasic binding site of Mrp1. While binding of ritonavir into the hydrophilic P-pocket of Mrp1 might stabilise and thereby stimulate the export of GSH that is bound to the hydrophobic H-Pocket, the export of larger substrates including GSSG or GS-B is inhibited due to the occupancy of the P-Pocket by ritonavir, since these larger substrates require binding to the P-Pocket in order to be transported (Fig. 7).

The here proposed mechanistic model for Mrp1-mediated transport processes combined new molecular *in silico* studies, which are in line with the findings of Johnson & Chen [12, 13], with experimental data for GSH export. Our new data are consistent with the proposed theoretical model of cooperative binding that predicted the presence of a bipartite binding site with different affinities of the individual pockets towards GSH and drugs for Mrp1-mediated export processes in cultured astrocytes [17]. Biochemical data confirmed that Mrp1 has two distinct binding sites for GSH or drugs that possess different affinities for either of both substrates and that binding of GSH subsequent to drug binding induces conformational changes of Mrp1 that facilitate export of the respective substrates [103].

Understanding the principles behind the transport mechanism of the ubiquitously expressed transport protein Mrp1, which is responsible for the export and detoxification of various types of (chemotherapeutic) drugs and potentially toxic xenobiotic substances with a broad variety of chemical characters, is crucial for predicting and understanding the occurrence of tolerance and/or side-effects of various types of drugs, especially in combinational prescription as it is currently the case for paxlovid. For instance, binding of the anticancer drugs doxorubicin and etoposide to the P-pocket of Mrp1, similar to ritonavir, would explain the reported transport inhibition of Mrp1 by these compounds and the simultaneous occurrence of cytotoxicity [100, 101]. And indeed, doxorubicin in addition to other anticancer drugs have very recently been shown in *in silico* docking experiments to bind to Mrp1 [104].

We would like to note that the observations and conclusions made in our study are most likely also applicable to transport processes mediated by human Mrp1, as both the amino acid sequence and the domain topology of rat and human Mrp1 show very high similarity. In the future, a more in-depth insight into the transport mechanism of various Mrp1 substrates and evaluation of multiple ligand binding poses with accurately determined binding free energies would be desirable. This requires computationally demanding enhanced-sampling MD techniques such as alchemical free energy calculations or funnel-shaped restrained metadynamics with Hamiltonian replica-exchange (fun-SWISH) [105], which are left out for further, more in-depth studies.

For the brain, a treatment with ritonavir might have severe consequences for the GSH metabolism, since it accelerates the export of GSH from astrocytes and neurons [52, 106]. But at least under conditions of optimal amino acid supply, GSH synthesis can fully compensate the ritonavir-induced acceleration of GSH export from astrocytes [52]. However, accelerated export of GSH results in increased extracellular GSH concentrations, which could have multiple consequences to neurons. On the one hand, GSH is hydrolysed by the astrocytic ectoenzyme γ-glutamyltransferase generating increased levels of the dipeptide CysGly and the neurotransmitter glutamate [39, 55, 107, 108] of which the latter is believed to induce excitotoxicity by overstimulation of glutamate receptors [109, 110]. On the other hand, an increased extracellular concentration of GSH might serve as precursor donor for neuronal GSH synthesis [107, 111]. Furthermore, ritonavir has been reported to induce oxidative stress [112, 113] as well as HIV infection itself [114–117]. Since astrocytes generate substantial amounts of GSSG during oxidative stress, which is subsequently released via Mrp1 to sustain the cellular thiol reduction potential [16, 39], a blockage of GSSG export through ritonavir in the brain of treated patients might therefore additionally reduce oxidative stress resistance of these cells. Therefore, chronic oxidative stress and GSH deficiency caused by HIV infection might be even more detrimental for brain cells under prescription of ritonavir due to its interference with Mrp1-mediated transport processes.

For the treatment of patients with ritonavir, the ability of ritonavir to enter the brain appears to be sufficiently high to decrease viral burden in the cerebrospinal fluid (CSF) [118], although the concentration of ritonavir in the CSF has been reported to be below 35 nM [119]. However, due to the high lipophilicity of ritonavir, the real tissue concentrations of ritonavir in brain are likely to be underestimated by CSF or plasma concentrations [120]. Whether the ritonavir concentrations in brain tissue after application of therapeutical doses are sufficiently high to affect Mrp1-mediated processes remains to be elucidated. In vivo studies are also required to investigate whether and how an application of therapeutical doses of ritonavir may modulate the GSH metabolism in brain as well as the supply of GSH precursors from astrocytes to neurons.

### Electronic Supplementary Material

Below is the link to the electronic supplementary material.

Supplementary Material 1
