# Supplementary material for: Modulation of Multidrug Resistance Protein 1-mediated Transport Processes by the Antiviral Drug Ritonavir in Cultured Primary Astrocytes
Source: Neurochem Res. 2023 Aug 21;49(1):66–84. doi: 10.1007/s11064-023-04008-5 (PMC10776481; doi:10.1007/s11064-023-04008-5)
Supplement: Supplementary file 1 — Supplementary Material 1 [file 11064_2023_4008_MOESM1_ESM.docx]

**Modulation of Multidrug Resistance Protein 1-mediated transport processes by the antiviral drug ritonavir**

**Christian Arend, Isabell L. Grothaus, Mario Waespy, Lucio Colombi Ciacchi and Ralf Dringen**

**Supporting Information**





**Figure S1: Extracellular LDH activity for experiments shown in Figure 1.** Astrocytes were incubated in the absence (a) or presence of 100 µM menadione (b) or 10 µM monochlorobimane (c) for the indicated time-points. The extracellular LDH activity was determined as marker for a potential loss in cell viability. The data shown represent means ± SD of values that had been obtained in experiments performed on three independently prepared astrocyte cultures. Statistical analysis of the significance of differences of the data obtained after incubation without (control) and with ritonavir was performed by one-tailed Student’s t-test. The levels of significance are indicated by ^#^p < 0.05.





**Figure S2:** **Extracellular LDH activity for experiments shown in Figure 2.** The cells were preincubated without (control) or with 100 µM BSO for up to 24 hours (a). Afterwards, the cells of the respective preincubation (control or BSO) were further incubated in the absence of BSO without or with 10 µM ritonavir for 3h (absence of ritonavir) or 1h (presence of ritonavir) (b). The extracellular LDH activity was determined as marker for a potential loss in cell viability. The data shown represent means ± SD of values that had been obtained from triplicates of one representative experiment.





**Figure S3: Extracellular LDH activity for experiments shown in Figure 3.** Astrocyte primary cultures were incubated for 30 min without or with 50 µM MK571, 30 µM ritonavir or 100 µM ritonavir in the absence (a) or the presence of 100 µM menadione (b) or 10 µM MCB (c). The extracellular LDH activity was determined as marker for a potential loss in cell viability. The data shown represent mean ± SD of values that had been obtained in experiments performed on three independently prepared astrocyte cultures. Significant differences of the data obtained after incubation without (control) compared to incubation with ritonavir or MK571 were analysed with ANOVA followed by the Bonferroni *post hoc*-test. The levels of significance are indicated by ^*^p < 0.05.


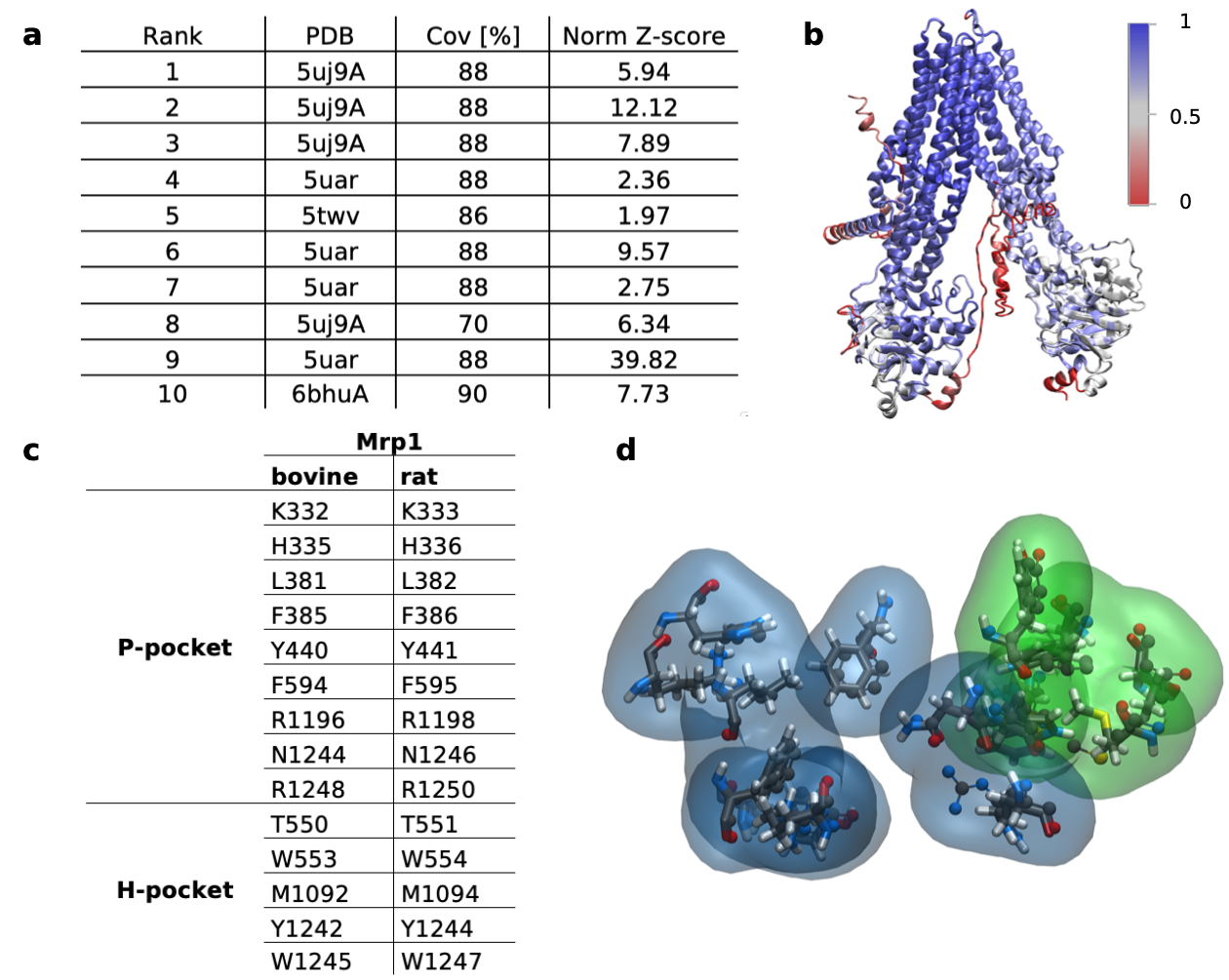


**Figure S4: Homology model generation for rat Mrp1 and its comparison with bovine Mrp1.** The i-Tasser algorithm based the model construction on template structures from bovine Mrp1 (pdb entry: 5UJ9 and 6BHU), cystic fibrosis transmembrane conductance regulator from zebrafish (pdb entry: 5UAR) and a potassium channel from rat (pdb entry: 5TWV) (a). The sequence similarity is described as coverage in % and the normalized Z-score evaluates the quality of alignment (z-score > 1 indicates good alignment) (Yang and Zhang, 2015). Structural alignment of rat and bovine (pdb entry: 5uj9) Mrp1, where structures are coloured by the Q_H_ value, a metric for structural homology/conservation, calculated with the VMD plugin MultiSeq (Roberts *et al.*, 2006) (b). A Q_H_ va­­lue of 1 implies that structures are identical, whereas low scores (0.1-0.3), represent a poor alignment. Amino acids predicted to be involved in binding are conserved sequence- (c) and structure-wise (d) between rat and bovine Mrp1 and can be separated into a positively charged cavity (P-pocket, green) and a hydrophobic part (H-pocket, blue). Amino acids in licorice (rat) or CPK (bovine) style, all color-labelled by their atom composition with carbon = cyan, nitrogen = blue, oxygen = red, hydrogen = white.

**Prediction of ligand binding positions**

Initial protein-ligand complexes were generated used the docking software AutoDock 4, describing here the most relevant findings. Docking parameters are given in the material and methods section of the manuscript. Estimated binding free energies indicated that GSH preferentially binds into the hydrophobic part of the binding pocket (H-pocket), although the hydrophilic pocket (P-pocket) only had a 0.8 kcal/mol lower binding free energy (Fig. S5a,b). In contrast, GSSG was 44 times out of 50 iterations docked to the P-pocket with the lowest binding free energy of -0.7 kcal/mol, whereas the H-pocket was predicted having a positive binding free energy (Fig. S5a,c). Docking analysis for ritonavir revealed its preference, binding to the P-pocket of rat Mrp1 with a higher affinity as reported for GSSG (Fig. S5a,d). Binding affinities reported for ritonavir binding to the H-pocket can be ignored, as no proper binding of ritonavir into the binding site was observed, but rather binding to underneath the H-pocket facing the cytosolic side. It needs to be noted that the standard error of the Autodock algorithm is known to be around ± 2-3 kcal/mol (Forli *et al.*, 2016). Therefore, here reported values (Fig. S5a) should be considered with caution and rather be interpreted as tendencies representing more probable binding modes.


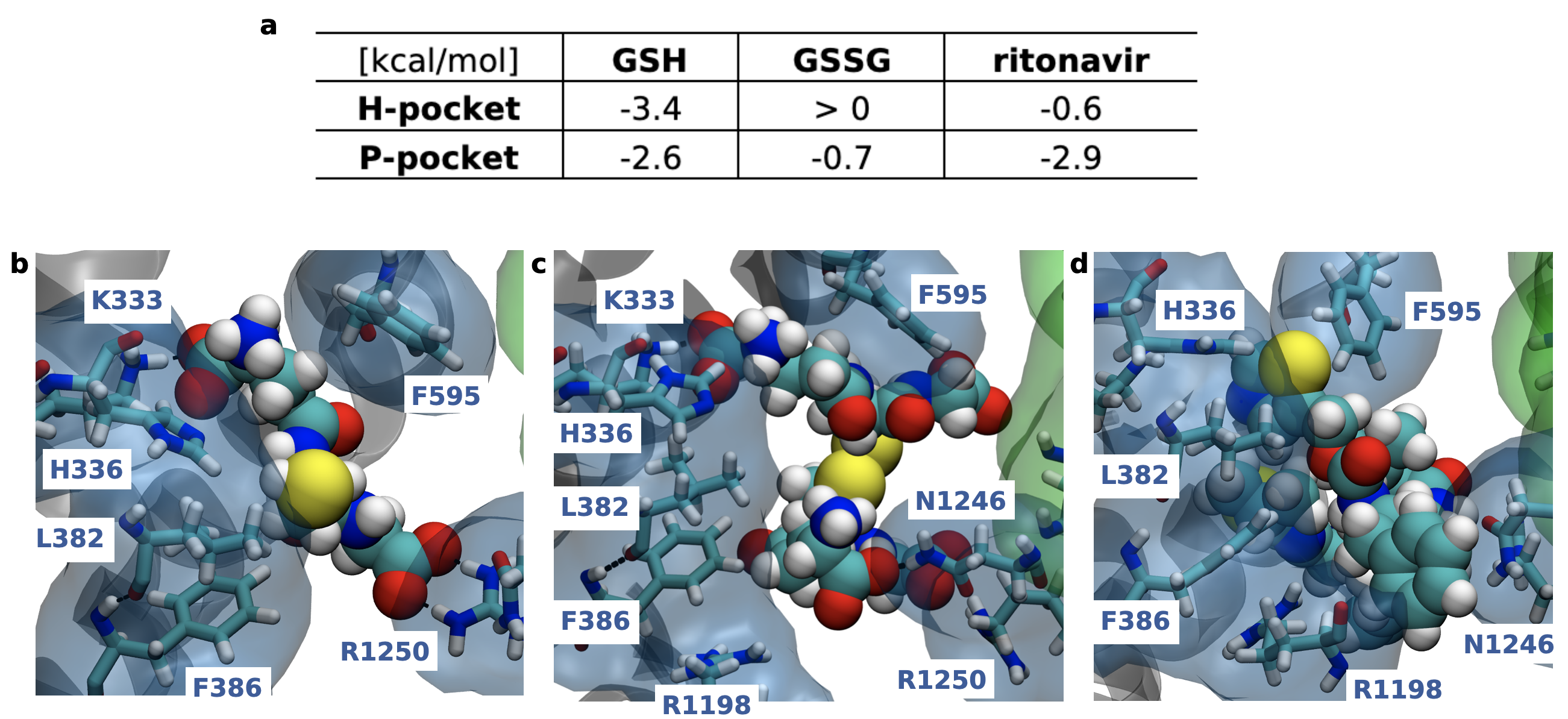


**Figure S5: Docking results of GSH, GSSG or ritonavir to rat Mrp1.** Lowest binding free energies [kcal/mol] measured for GSH, GSSG or ritonavir during docking analysis to the P- and H-pocket of rat Mrp1 (a). Binding positions of GSH (b), GSSG (c) or ritonavir (d) to the P-pocket of rat Mrp1, captured after minimization and equilibration of docked complexes. Volume surface of the P-pocket is represented in a transparent blue. GSH, GSSG and ritonavir in VDW style and amino acids in licorice style, all color-labelled by their atom composition with carbon = cyan, nitrogen = blue, oxygen = red, hydrogen = white.


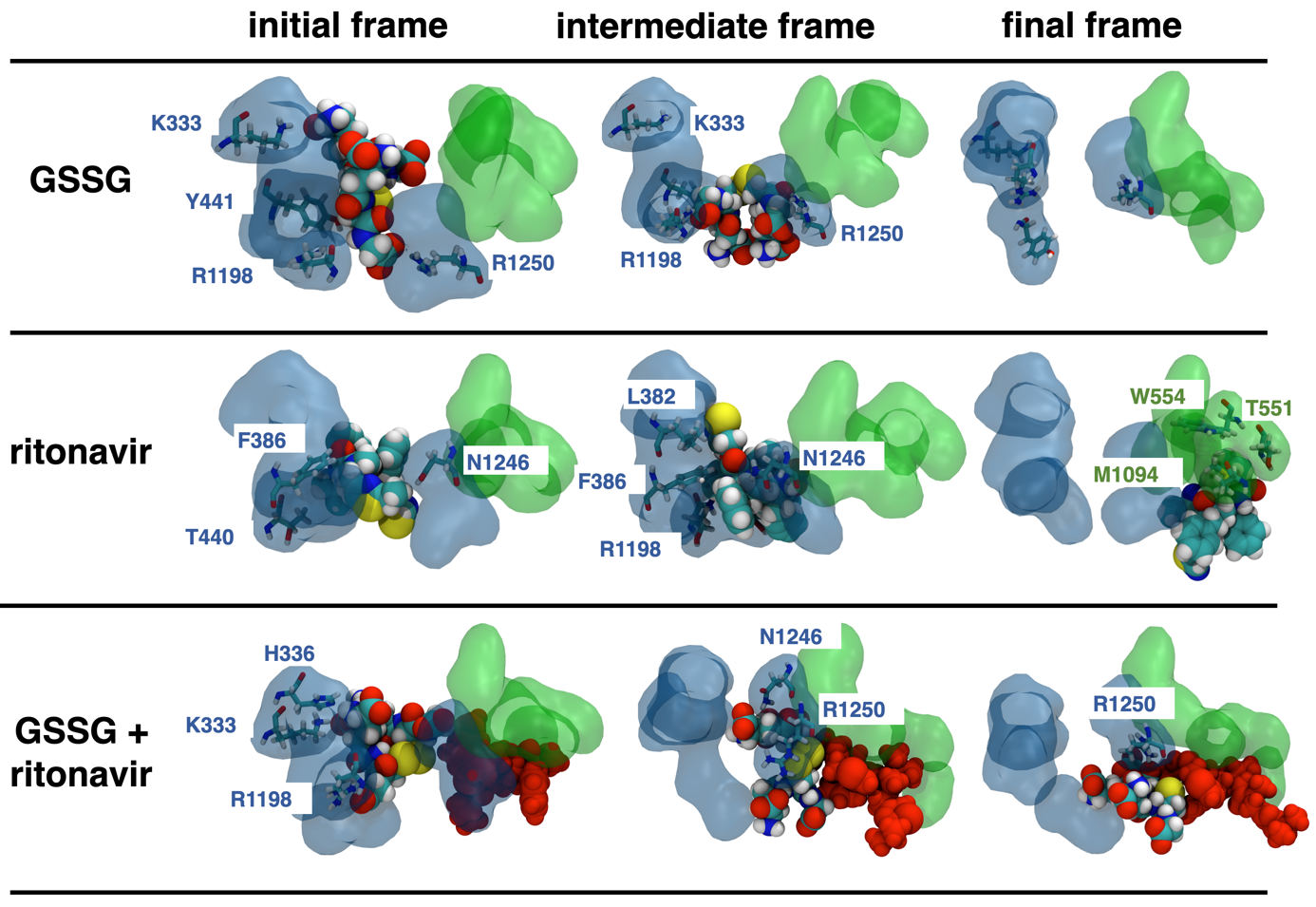


**Figure S6: Representative ligand binding poses of GSSG, ritonavir and GSSG plus ritonavir within the binding pocket of rat Mrp1 observed in MD simulations of 1 µs.** The initial frame, an intermediate frame with key interactions and the final frame of each simulation is shown. Binding of GSSG was only observed to the P-pocket, however forming the same interaction as a single GSH molecule with amino acids of the binding site (K333, Y441, R1198, R1250). Ritonavir was stably binding to the P-pocket of rat Mrp1 for 700 ns before diffusing below the H-pocket facing the cytosol. Simultaneous binding of GSSG and ritonavir (red) could not be observed, as both compounds did not fit together into the binding site. Volume surfaces of the P-pocket and H-pocket are represented in a transparent blue and green, respectively. Amino acids are colour-labelled by their atom composition with carbon = cyan, nitrogen = blue, oxygen = red, hydrogen = white. Ritonavir molecules in combination with GSSG are labelled in red. Only amino acids close to the ligand where explicitly shown. Lowest binding free energy structures were selected as starting formations from the ligand docking experiments. Movies of the trajectory progression can also be found under https://doi.org/10.5281/zenodo.6592231 for all systems.


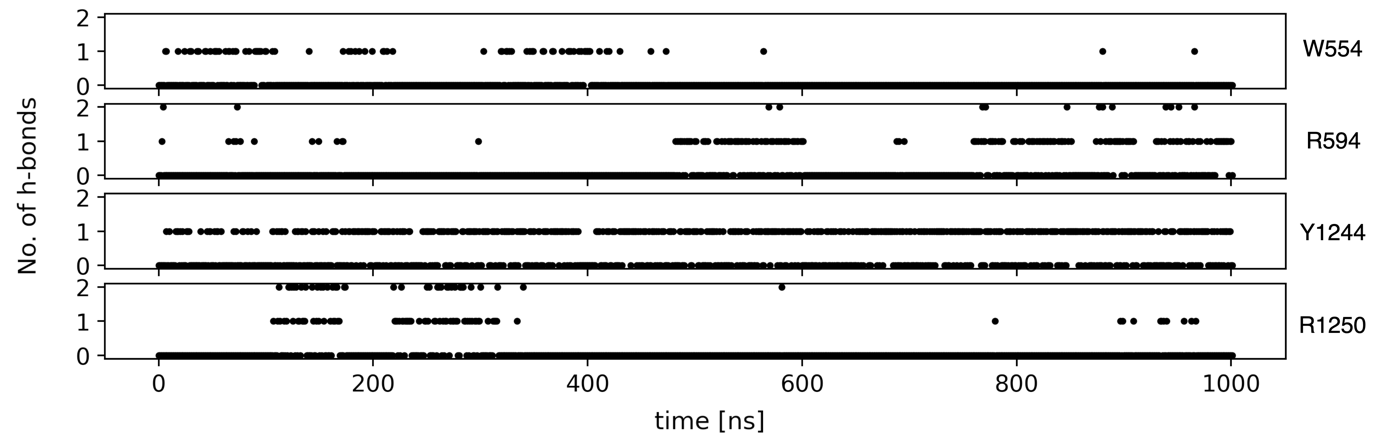


**Figure S7: Occurrence of hydrogen bonds between GSH and the binding site of Mrp1.** Number of hydrogen bonds between GSH and residues W554, R594, Y1244, R1250 measured during the simulation of GSH + ritonavir binding to rat Mrp1, respectively, as supporting information for Figure 6 showing the stabilizing effect of ritonavir on the binding of GSH to rat Mrp1.


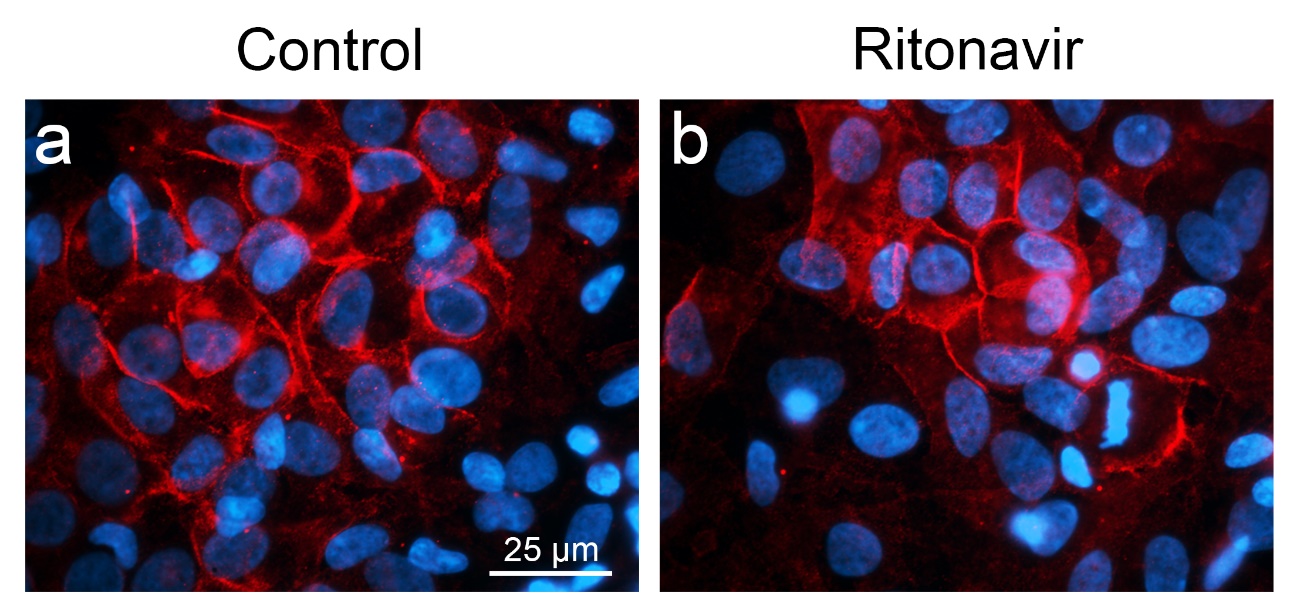


**Figure S8: Expression of Mrp1 in astrocyte cultures.** Immunocytochemical detection of Mrp1 (red) was performed after the cells were incubated in absence (a) or presence of 10 µM ritonavir (b) for 3 h. Additionally, the nuclei were stained with DAPI (blue). The scale bar in panel a applies to both panels. The primary rat anti-MRPr1 antibody was obtained from Enzo (Lörrach, Germany) and the Cy3-coupled secondary donkey anti-rat immunoglobulin G (IgG) antibody was obtained from Dianova (Hamburg, Germany).
